# Slaveholder ancestry and current net worth of members of the United States Congress

**DOI:** 10.1371/journal.pone.0308351

**Published:** 2024-08-21

**Authors:** Neil K. R. Sehgal, Ashwini R. Sehgal

**Affiliations:** 1 Department of Computer and Information Science, University of Pennsylvania, Philadelphia, Pennsylvania, United States of America; 2 Population Health and Equity Research Institute, Institute for HOPE, The MetroHealth System, Case Western Reserve University, Cleveland, Ohio, United States of America; Iowa State University, UNITED STATES OF AMERICA

## Abstract

**Background:**

Whether and how much past slavery affects contemporary social and economic conditions in the United States is an area of active debate. Newly available data on which members of the United States Congress are descendants of slaveholders provides an opportunity to examine this topic. This study sought to determine the relationship between slaveholder ancestry and net worth among members of Congress.

**Methods:**

Total assets and liabilities were collected from financial declarations of all members of Congress as of April 15, 2021. Net worth was estimated as the difference between total assets and liabilities. Information on slaveholder ancestry was obtained from a Reuters investigative series based on an extensive review of historical documents and verification by board-certified genealogists. Quantile regression was used to determine the association between net worth and slaveholder ancestry after adjustment for demographic factors.

**Results:**

The median net worth of the 535 members of Congress was $1.28 million (interquartile range $0.11–5.87 million). On univariate analysis, net worth was associated with increased age, White race, increased education, and number of individuals enslaved by ancestors. On multivariate analysis, net worth was independently associated with age, White race, and number enslaved. Legislators whose ancestors enslaved 16 or more individuals had a $3.93 million (95% confidence interval 2.39–5.46) higher net worth compared to legislators whose ancestors were not slave owners after adjustment for age, sex, race, ethnicity, and education.

**Conclusions:**

Past slaveholding practices are independently associated with current wealth among members of Congress. Because members of Congress are a highly selected group, further work is needed to understand how slaveholder ancestry affects current wealth in the general population to inform efforts to reduce social and economic disparities.

## Introduction

Whether and how much past slavery affects contemporary social and economic conditions in the United States is an active area of debate. Many slaveholders were able to recover financially despite emancipation of their slaves after the Civil War (1861–1865) [1]. They did this in part by replacing slavery with convict leasing and sharecropping, voting disenfranchisement, and legal discrimination (Jim Crow), all of which persisted into the 20th century. Most Americans believe slavery continues to influence the position of Black people in society today, although the proportion expressing this view differs greatly by race, ethnicity, and political affiliation [2]. Several ecological analyses have linked the intergenerational effects of slavery to a variety of outcomes, including inequality, poverty, education, voting behavior, and life expectancy [3–6]. However, no studies have determined the impact of having a slaveholder vs. a non-slaveholder ancestor on current wealth at an individual level.

Conducting an individual-level analysis would require the ability to trace ancestry from 1865 to the present, information on current wealth and relevant covariates, and an adequate sample size. A recent rigorous analysis by Reuters identified 100 members of the United States Congress who are descendants of slaveholders and quantified the number of individuals enslaved by their ancestors [7]. Connecting this information to financial reports that legislators file annually provides an opportunity to explore the current impact of past slaveholding practices. We therefore sought to determine the relationship between slaveholder ancestry and net worth among members of Congress.

## Methods

We collected demographic, financial, and slaveholder ancestry information as of April 15, 2021 for all members of the 117th Congress. If a seat was vacant in April 2021, we used data for the previous office holder.

### Demographic data

Demographic variables, including age, sex, race, ethnicity, and education, were obtained initially from Daily Kos’ 117th Congress Guide [8]. We then manually validated each member’s demographic information against their Congressional website. If any demographic data elements were not available, we reviewed each member’s Wikipedia page [9].

### Financial data

Members of Congress are required to file publicly available financial disclosures annually. Specific assets and liabilities of legislators for the year 2020 were obtained initially from the “Conflicted Congress” project by Business Insider [10]. This database compiled publicly available financial disclosures, listing each member’s assets and liabilities in a machine-readable format. We manually validated this information by examining individual assets and liabilities for a randomly selected 10% of the sample. In addition, Business Insider did not collect information on several members who had lengthy handwritten disclosures. For these members, we manually reviewed these handwritten disclosures [11, 12].

Net worth estimation followed the methodology laid out by OpenSecrets, a nonprofit campaign finance group [13]. Legislators report the value of each asset and liability within a specific range, such as $1,001 - $15,000 or $15,001 - $50,000. We summed the maximum and minimum values separately in order to calculate the maximum and minimum for total assets and liabilities. Maximum net worth was then calculated as the maximum total assets minus the minimum total liabilities, and vice versa for the minimum net worth. The midpoint of this range served as our primary estimate of each legislator’s net worth.

### Slaveholder ancestry

Information on slaveholder ancestry was sourced from Reuters’ investigative series “Slavery’s Descendants” which covered members of Congress as of April 15, 2021 [7]. For each member, Reuters assessed direct descent from slaveholders through searches of census documents, genealogy websites, and publicly available records such as wills; birth, marriage and death certificates; grave and cemetery records; digitized state archives; news articles; biographical books; and public record requests. Reuters limited its investigation to direct lineal ancestors of members, and only considered evidence of slaveholding in the United States after 1776. As a result, neither slaveholding in the United States before 1776 nor slaveholding outside of the United States at any time were included. Each case was then verified by two board-certified genealogists. In instances where multiple slaveholding ancestors were identified, the reported number enslaved pertained to the ancestor with the highest number of enslaved individuals.

### Statistical analysis

We used descriptive statistics (mean, standard deviation, percent, median, interquartile range) to summarize the data. We used the Mann-Whitney U-test, the Kruskal-Wallis test, and Kendall’s tau coefficient to examine the univariate relationship between net worth and dichotomous (sex), nominal (race/ethnicity), and continuous or ordinal (age, education, number enslaved by ancestor) predictor variables, respectively. Given the skewed distribution of net worth, we used median regression (quantile regression) to estimate the independent association between median net worth and slaveholder ancestry after controlling for age, sex, race/ethnicity, and education. To discern potential non-linear relationships, we categorized age, education, and number enslaved into discrete groups. We categorized members of Congress with slaveholder ancestry into three roughly equal sized groups based on number enslaved and created another category for members without slaveholder ancestry. Quantile regression standard errors were calculated using the Bofinger bandwidth method [14]. We also performed five ancillary analyses. First, wealthy families can provide more educational opportunities to their children, and higher education may lead to increased wealth. As a result, we conducted analyses that excluded education as a predictor variable. Second, virtually all legislators with slaveholder ancestors were White. As a result, we conducted separate analyses limited to White legislators. Third, we performed quantile regression at the 75th percentile to examine predictor variables associated with being in the top quartile of net worth among the entire sample. Fourth, we repeated analyses using maximum and minimum estimates of net worth. Fifth, we explored the sensitivity of our results to varying the cut point for the largest category of slave holders. All analyses were conducted using JMP version 17.0.0 (SAS Institute, Cary, North Carolina).

## Results

The characteristics of the 535 legislators (435 representatives, 100 senators) are listed in Table 1. Their average age was 59.7 years, about three fourths were men, and about three fourths were White. Their median net worth was $1.28 million. Of all legislators, 131 (24.5%) had a net worth <$100,000 and 89 (16.6%) had a net worth >$10 million. A total of 100 legislators (18.7%) were descendants of slaveholders.

**Table 1:** Characteristics of legislators in 117th congress (n = 535).

Mean age, years (standard deviation)	59.7 (11.9)
Sex	
Male	391 (73.1%)
Female	144 (26.9%)
Race/Ethnicity	
White*	410 (76.6%)
Black*	56 (10.5%)
Hispanic**	46 (8.6%)
Other*	23 (4.3%)
Education	
High school or associate degree	26 (4.9%)
Undergraduate degree	142 (26.5%)
Graduate degree	367 (68.6%)
Slaveholder descendant	
No	435 (81.3%)
Yes	100 (18.7%)
Number enslaved by ancestor	
None	435 (81.3%)
1–5	42 (7.9%)
6–15	31 (5.8%)
16 or more	27 (5.0%)
Median net worth, $ million	1.28 (0.11–5.87)
(interquartile range)	

*Non-Hispanic

**Any race

On univariate analysis, net worth was associated with increased age, White race, increased education, and number of individuals enslaved by ancestors (Table 2). For example, the median net worth was $0.52 million for legislators younger than 55 years old and $2.64 million for legislators 65 years and older. On multivariate analysis, net worth was independently associated with age, White race, and number enslaved (Table 2). Legislators whose ancestors enslaved 16 or more individuals had $3.93 million higher net worth compared to legislators whose ancestors were not slave owners after adjustment for age, sex, race, ethnicity, and education.

**Table 2:** Univariate and multivariate relationship between legislator characteristics and net worth (n = 535). Bolded multivariate results are statistically significant.

	n	Median net worth, $ million	Univariate	Multivariate regression estimate,
			p value	$ million (95% confidence interval)
Age, years			< .001	
<55	162	0.52		Reference
55–64	173	1.22		0.27 (-0.58, 1.11)
≥65	200	2.64		**1.67 (0.85, 2.49)**
Sex			0.51	
Male	391	1.09		Reference
Female	144	1.69		0.48 (-0.28, 1.24)
Race/Ethnicity			< .001	
White	410	1.81		Reference
Black	56	0.06		**-1.33 (-2.45, -0.21)**
Hispanic	46	0.16		-1.15 (-2.37, 0.07)
Other	23	2.39		-0.003 (-1.67, 1.66)
Education			.04	
High school or associate degree	26	0.31		Reference
Undergraduate degree	142	0.91		-0.09 (-1.73, 1.54)
Graduate degree	367	1.37		0.17 (-1.38, 1.72)
Number enslaved by ancestor			.003	
None	435	1.11		Reference
1–5	42	2.59		0.14 (-1.11, 1.40)
6–15	31	2.87		0.95 (-0.49, 2.39)
16 or more	27	5.62		**3.93 (2.39, 5.46)**

Multivariate analysis results were similar if education was excluded as a predictor variable. Regression estimates were 0.20 (95% confidence interval -1.07 to 1.47) for 1–5 slaves, 0.94 (-.52 to 2.40) for 6–15 slaves, and 3.91 (2.36–5.47) for 16 or more slaves compared to no slaves. Analyses limited to White legislators (Table 3) also had similar results. White legislators whose ancestors enslaved 16 or more individuals had $3.41 million higher net worth compared to White legislators whose ancestors were not slave owners after adjustment for age, sex, and education. Quantile regression estimates at the 75th percentile were -0.13 (-5.09 to 4.82) for 1–5 slaves, 1.76 (-3.92 to 7.45) for 6–15 slaves, and 4.41 (-1.65 to 10.46) for 16 or more slaves compared to no slaves. Multivariate analyses based on maximum or minimum net worth estimates yielded similar findings as analyses based on our primary midpoint estimate, i.e. that legislators whose ancestors enslaved 16 or more individuals had a statistically significantly higher net worth compared to legislators whose ancestors were not slave owners. Varying the cut point for the largest category of slaveholders across the range from 13 to 19 slaves also yielded similar findings. Multivariate regression estimates for the largest category of slaveholders were 3.62 (2.25, 5.00) for 13 or more slaves, 3.65 (2.22, 5.07) for 14 or more slaves, 3.65 (2.16, 5.14) for 15 or more slaves, 4.81 (3.24, 6.38) for 17 or more slaves, 4.31 (2.69, 5.94) for 18 or more slaves, and 4.80 (3.15, 6.45) for 19 or more slaves.

**Table 3:** Univariate and multivariate relationship between legislator characteristics and net worth among white members of congress (n = 410). Bolded multivariate results are statistically significant.

	n	Median net worth, $ million	Univariate	Multivariate regression estimate,
			p value	$ million (95% confidence interval)
Age, years			< .001	
<55	110	0.77		Reference
55–64	146	1.42		0.44 (-0.84, 1.72)
≥65	154	3.50		**2.84 (1.57, 4.10)**
Sex			.16	
Male	317	1.47		Reference
Female	93	2.71		1.09 (-0.11, 2.28)
Education			.37	
High school or associate degree	18	0.67		Reference
Undergraduate degree	109	2.64		0.47 (-2.11, 3.05)
Graduate degree	283	1.81		0.66 (-1.80, 3.11)
Number enslaved by ancestor			.15	
None	312	1.57		Reference
1–5	40	2.59		-0.003 (-1.71, 1.70)
6–15	31	2.87		0.50 (-1.41, 2.41)
16 or more	27	5.62		**3.41 (1.38, 5.45)**

## Discussion

We found that members of the United States Congress whose ancestors had 16 or more slaves have a current net worth that is five-fold larger than legislators whose ancestors were not slave owners. This sizeable difference in net worth persisted even after adjustment for potential confounders such as age, sex, race, ethnicity, and education and in ancillary analyses limited to White legislators. To our knowledge, this study is the first to examine individual level associations between slaveholder ancestry and net worth in the present day. Other strengths of the study include availability of data on specific assets and liabilities, rigorous review of historical documents to identify slaveholder ancestry, and inclusion of data on the number of slaves owned in the past.

Our findings on the impact of slaveholder ancestry on wealth are consistent with previous research on the topic, much of which was done at the county level. For example, studies have found counties with higher rates of slavery in 1860 are associated with higher contemporary levels of racial inequality in education, as well as better socioeconomic outcomes (e.g. income, home ownership, food security) among Whites [3, 4]. Other work finds counties with wealthier slaveowners before emancipation were associated with lower economic development that persisted through 1950. This association is attributed, in part, to the enduring political influence wielded by slaveowners and their reluctance to support widespread educational initiatives [15]. Recent advances in linking digitized census records have allowed analysis of data at the individual level. For instance, a study examined White Southern households with large numbers of slaves in 1860 and a comparison group of equally wealthy households that had few slaves and instead owned more land, livestock, buildings, and other assets. By 1900, the sons of larger slaveholders had almost recovered in occupation-based wealth, and by 1940, grandsons of larger slaveholders completely recovered in both annual earnings and educational attainment. Due to data limitations, the researchers were unable to examine grandsons’ wealth in 1940 [16]. In addition, the association we observed between legislators’ net worth and increased age, White race, and more education follows the pattern seen across the American population at large [17, 18].

Wealth and privilege may be transmitted across multiple generations through a variety of mechanisms. Inheritance laws and related policies such as low estate taxes and mechanisms to create trusts allow for wealth perpetuation. Access to social networks of other wealthy individuals, opportunities to attend prestigious educational institutions, and entry into prominent occupations provide additional benefits [19]. Wealthy families are also able to hire professionals to manage their estates, trusts, and other investments. Moreover, wealthy individuals use their political influence and philanthropic giving to exert influence on regulations and tax policies and on public perceptions [19]. The study of White Southern households mentioned above found that social connections and marriages to other elite families explained larger slaveholding families’ rapid recovery, not their abilities or entrepreneurial skills [16]. In addition, the name recognition that results from being part of an established family can be helpful when competing in political elections [20]. Our results, over 150 years after emancipation, provide further evidence for the durability of wealth across generations. It is worth emphasizing that wealth from any source (whether slavery is involved or not) generally creates intergenerational benefits. Wealthy individuals accrue political power which they use to further enhance their wealth in a positive feedback loop [21].

Several limitations must be considered in interpreting the study results. First we focused on members of Congress because of the availability of reliable data on slaveholder ancestry and current net wealth for this group. But members of Congress are wealthier than the general population, so our analyses condition to some extent on the outcome variable of net wealth. This approach may introduce associations that do not exist in the general population and is referred to as endogenous selection bias or collider bias [22–25]. However, many members had a modest net wealth (about one-fourth with a net wealth <$100,000) and our findings are consistent with several previous studies in the United States and Europe about intergenerational transfer of wealth and privilege [16, 19, 26–32]. Nevertheless, we must exercise caution in extrapolating the results of our study to all Americans (or even to politicians who are not elected to Congress). Second, the Reuters analysis was restricted to slavery after the founding of the United States and excludes those who may be descendants of slaveholders prior to 1776. It is worth noting that no legislator has contested Reuters’ findings regarding their ancestral ties to slaveholders [7]. Third, there are several aspects of legislators’ personal financial disclosures which may impact our net worth calculation. Federal retirement accounts, personal residences that do not produce income, and some types of personal property are not reported [13]. Additionally, the reporting of large assets is provided in broad ranges, making it difficult to precisely assess net worth. Fourth, our modest sample size resulted in wide confidence intervals that may have limited our ability to identify a dose-response relationship between number of slaves and net worth. Due to the limited sample size, we are also unable to determine a precise threshold number of slaves owned by ancestors that correlates with an increase in current net wealth. Fifth, our data do not permit any mechanistic or causal interpretations. Additional work is needed in this area, e.g. to understand the role of political power and occupational paths in intergenerational wealth transfer. There may also be other channels that connect past and current wealth. For example, geographic locations in the southern United States where slavery was prevalent may have economic development trajectories that affect wealth transmission independent of slaveholding.

In conclusion, we find evidence which suggests that slaveholding in the past may continue to affect certain individuals today. While members of Congress do not bear personal responsibility for the actions of their ancestors, further work is needed to understand how slaveholder ancestry affects current wealth in the general population to inform efforts to reduce social and economic disparities.
